# Establishing the Convergent Validity of the Travel Habit Questions in the Health Behavior in School-Aged Children Questionnaire by Quantifying Active Travel in Norwegian Adolescents

**DOI:** 10.3389/fspor.2022.761723

**Published:** 2022-04-06

**Authors:** Lena Malnes, Tommy Haugen, Bjørge Herman Hansen, Elin Kolle, Sveinung Berntsen

**Affiliations:** ^1^Department of Sport Science and Physical Education, University of Agder, Kristiansand, Norway; ^2^Department of Sports Medicine, Norwegian School of Sport Sciences, Oslo, Norway

**Keywords:** validation, agreement, travel diary, logs, active commuting, travel habits, cycling, walking

## Abstract

**Background:**

Active travel (cycling or walking to school) can be a substantial part of adolescents' daily physical activity. Research on transport activities primarily relies on self-reported indices of travel mode and travel time. However, many researchers do not report the psychometric properties of their instruments. The Health Behavior in School-aged Children (HBSC) questionnaire is a commonly used instrument, but the items in this questionnaire on travel habits have not yet been validated. The present study was conducted to investigate the convergent validity and agreement between the HBSC items and a travel diary on (1) transport mode to and from school and (2) travel time to school.

**Methods:**

The study sample consisted of 50 participants in the 9th grade (15 ± 0.3 years, 62% girls) from seven Norwegian schools. Outcome variables included transport mode and travel time derived from the HBSC items and a five-day travel diary. Convergent validity was assessed by evaluating Cohen's kappa for travel mode and the correlation coefficient (Spearman Rho) for travel time. Simple agreement calculations between the two measurement methods were also conducted.

**Results:**

The association between the HBSC questionnaire and the diary for travel mode to and from school was κ = 0.63 (*P* < 0.001) and κ = 0.77 (*P* < 0.001), respectively. The total agreement between the HBSC questionnaire and the diary for was 78%. However, the agreement was higher for walking (88%) and cycling (91%) than for motorized transport (67%). For travel time, the Spearman correlation coefficient was ρ = 0.60 (*P* < 0.001) between the HBSC questionnaire and the diary. The total agreement on travel time was 67%; however, active commuters (86%) seemed to more accurately estimated travel time than motorized commuters (55%).

**Conclusion:**

Although the overall agreement between the HBSC questionnaire and the diary for mode of transport was 78%, the HBSC questionnaire may underestimate the prevalence of motorized transport compared to walking and cycling.

**Trial Registration:**

ClinicalTrials.gov, identifier: NCT03817047.

## Introduction

Although regular physical activity has health benefits (Warburton et al., 2010), most Europeans do not meet the current international guidelines for physical activity, even at a young age (Steene-Johannessen et al., 2020), and inactivity remains a significant public health challenge (Kohl et al., 2012). Active travel (cycling or walking to and from school) can be an effective way to incorporate physical activity into everyday life (Sahlqvist et al., 2012; Yang et al., 2014), and efforts to facilitate active travel can help address the concern of physical inactivity.

In the last decades, an increasing number of studies have been conducted on active travel among children and adolescents (Chillón et al., 2011; Villa-González et al., 2018). Chillón et al. (2011) reviewed interventions to increase the prevalence of active travel; and found that school-based interventions had a small effect. However, only two of the included studies reported data on the validity of their measurement instruments, leaving uncertainty regarding the accuracy of their findings. Seven years later, Larouche et al. published an updated review, based on a replicated search, and rated most studies to be of poor quality, partly due to the lack of psychometric properties indicating the validity of their measurements (Larouche et al., 2018). Therefore, future studies with valid measures on travel habits are required (Chillón et al., 2011; Lubans et al., 2011; Lu et al., 2014; Larouche et al., 2018).

Research on travel behavior has mostly relied on self-reported indices of measurement (Chillón et al., 2011; Herrador-Colmenero et al., 2014; Larouche et al., 2018). However, many researchers do not report validity data on their questionnaire items and diaries. Herrador-Colmenero et al. (2014) reviewed 158 articles mentioning self-reported measures to assess active travel and found that only eight articles included data on validity. Criterion validity has been defined as “the extent to which a research instrument is related to other instruments that measure the same variables,” and convergent validity measures the correlation with similar instruments (Heale and Twycross, 2015). Observation through a camera is one way to identify and validate modes of transport (Kelly et al., 2014; Carlson et al., 2015); it can provide objective data and is therefore thought to be more accurate than self-reported data (Doherty et al., 2013; Kelly et al., 2014; Carlson et al., 2015). However, the use of a camera can increase participant burden and raise ethical concerns regarding the privacy of children and adolescents (Everson et al., 2019). Other researchers have applied parental questionnaires (Evenson et al., 2008; Herrador-Colmenero et al., 2014; Larouche et al., 2017) and self-reported diaries (Petrunoff et al., 2013) to validate questionnaire items on travel habits in young individuals.

The great diversity in self-reported items on active travel (Chillón et al., 2011; Herrador-Colmenero et al., 2014; Lu et al., 2014) can make it difficult to draw comparisons between studies. The Health Behavior in School-aged Children (HBSC) questionnaire contains items to measure active travel (Roberts et al., 2009). It is a widely used instrument to collect data on travel mode and duration (Gropp et al., 2012, 2013; Helmerhorst et al., 2012; Loureiro and Gaspar, 2014; Morgan et al., 2016; Yang et al., 2016; Hollein et al., 2017; Ian et al., 2017; Pavelka et al., 2017). To our knowledge, data on the convergent validity of the transport-related items included in the HBSC questionnaire have not been published.

The present study was conducted to investigate the convergent validity and agreement between the HBSC questionnaire and a travel diary on (1) mode of transport to and from school and (2) travel time to school among students aged 14–15 years.

## Materials and Methods

### Study Design

The present study was a part of the School in Motion (ScIM) project (Solberg et al., 2021), a cluster-randomized controlled multi-center study conducted in the school year 2017/18. Pupils in the 9th grade (14–15 years) from different parts of Norway had previously participated in pre- and mid-data collection phases. The present study comprised participants from the county of Agder at post-test. The participants filled out a five-day travel diary and reported travel habits in a questionnaire during spring 2018.

### Study Participants

Overall, 341 participants were invited to participate in the study by filling out a travel diary, in addition to the original test battery in the main project. Of the 341 pupils invited, 57 returned the travel diary with sufficient data on travel mode to and from school (*n* = 54 and 52, respectively) and data on travel time (*n* = 49) to school. Of these, seven participants did not answer the questionnaire, resulting in 50 participants reporting data on travel mode (47 and 46 to and from school, respectively) and travel time (*n* = 43) to school.

### Measures

#### Demographic Variables

Data on age, gender, and ethnicity were self-reported. We also measured the participants' height, weight, and waist circumference.

#### Travel Diary

A member of the research team handed out the travel diary and explained how to report the data. The reporting started the next day for 5 consecutive weekdays. In the diary, the participants reported whether they traveled by foot, by bicycle, e-bike, by car, collectively or by other. The participants also noted the time they traveled from home to school, arrived at school, traveled from school back home, and arrived home after school. If needed, the participants could also write a comment. The travel diary was distributed between April and June on various dates to avoid holidays and special events at the schools. Opening hours for the schools were between 8 and 9 am, and closing hours varied between 1 and 3 pm, varying between schools and day of the week. The participants registered travel data between 3 and 36 days before answering the questionnaire, except at one school completing 9 days after answering the questionnaire.

The most-reported mode of transport in the diary was considered the participants' typical mode of transportation and included in the analysis. In cases where the typical mode of transport was unclear (*n* = 4), travel mode was set to missing.

For travel time, the median travel time (minutes) of all logged days was defined as the typical travel time and included in the analysis. When traveling to school in the morning, the participants reported departure time (hh:mm) from home and time of arrival to school. We calculated travel time by subtracting the time of arrival to school from the time of departure. Some participants provided an approximate timeframe in their diary; for example, left for school (hh:mm) 08:10-08:15. In these cases, the mid-point (for example, 08:12:30) was used to calculate the travel time.

#### HBSC Questionnaire Items

We retrieved three questionnaire items from the HBSC questionnaire (Roberts et al., 2009); two measuring modes of transport (to and from school) and one measuring travel time to school. Due to large weather variations in the Nordic countries during a year (Kolle et al., 2009), there is a risk of estimation bias, which we addressed by adding seasonal and monthly specifications (summer and winter half of the year) to the HBSC items. The items measuring modes during the summer season were included in the present study. More specifically, the question states, “On a typical day in the spring/summer (April to September) is the main part of your journey to school made by…?” followed by a question capturing travel mode from school. The participants answered with the following options: by walking, by bicycle, by car/motorcycle/moped, by bus/trains/subway/ferry, or by other. No participants traveled by car from school, and 10 traveled by car to school, according to the diary. Therefore, simple agreement calculations on car and bus were performed separately, but pooled as motorized transport when presenting results, for ease of communication. The participants reported travel time by answering “How much time does it usually take you to get from home to school in the summer?” with one of the following responses: <5, 5–15, 15–30, 31 min to 1 h, and more than 1 h.

### Statistical Analyses

The descriptive data are presented as percentage of participants (N), mean (SD), and median (IQR) for categorical, normally distributed, and skewed data, respectively. Differences in demographic data between the included sample and the invited sample were analyzed by the chi-square, independent samples *t*-test, or Mann-Whitney *U*-test as appropriate. The convergent validity of the HBSC questionnaire in relation to the travel diary was assessed by Cohens Kappa (κ) on travel mode and Spearman correlation (ρ) on travel time. The percentage agreement between the two instruments was determined by simple calculations. Data were analyzed using Statistical Package Social Sciences, SPSS version 22 (Chicago, Illinois). The significance threshold was set at *p* < 0.05.

## Results

### Descriptive Statistics

**Figure 1:**
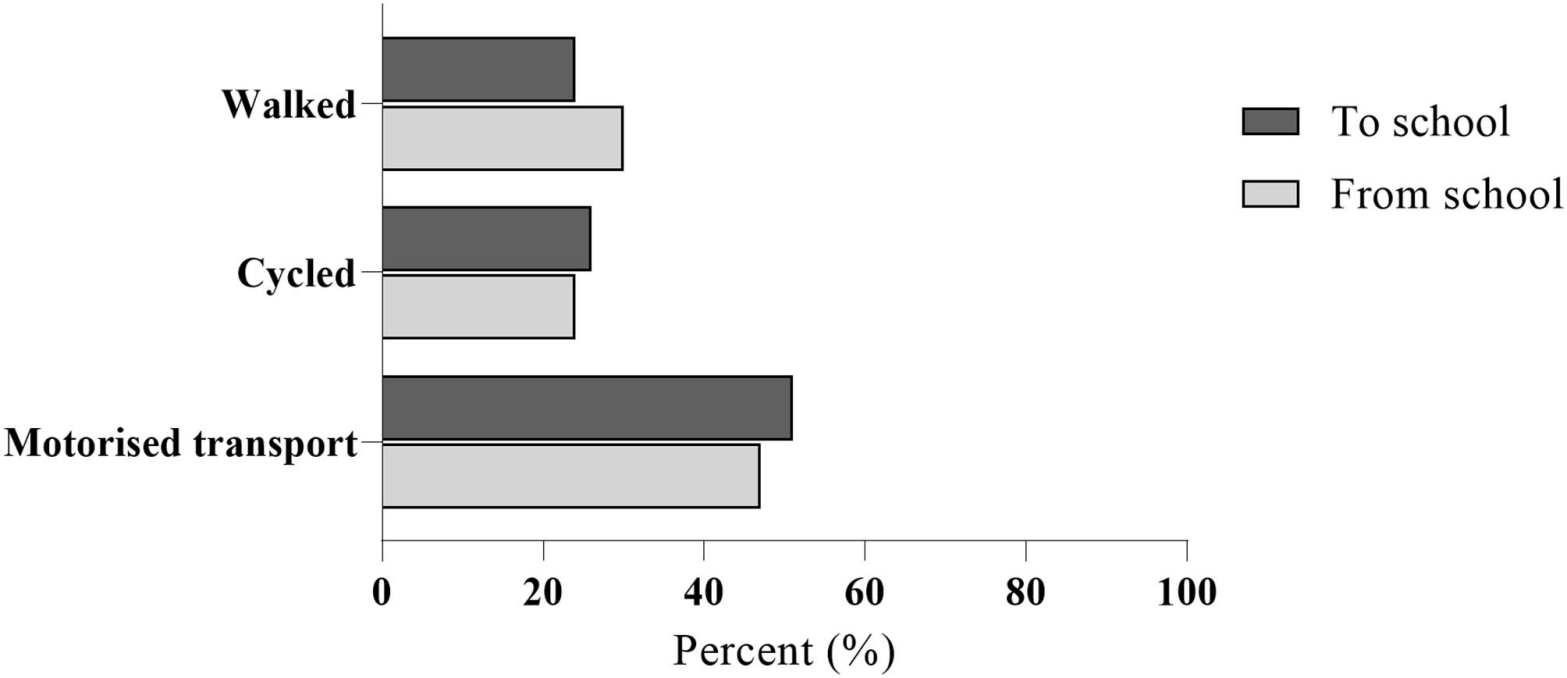
Percentage (%) of participants to (*n* = 47) and from (*n* = 46) school divided by means of transport from travel diaries.

No significant differences were observed between the included sample and the total invited sample (Table 1). Most participants typically traveled by motorized transport (48%), followed by walking (27%), and cycling (25%), as illustrated in Figure 1. The median (IQR) travel time to school was 15 (15) minutes.

**Table 1:** Characteristics of the sample and the main population.

	**Included sample**	**Invited sample**
	**(*N* = 50)**	**(*N* = 291)**
Girls, *n* (%)	31 (62)	147 (51)
Immigrant mother or father, *n* (%)	6 (12)	50 (23)
Age years, mean (SD)	15 (0.3)	15 (0.3)
Height cm, mean (SD)	171 (6.8)	170 (8.0)
Weight kg, median (IQR)	59 (11.8)	59 (13.5)
Waist circumference cm, median (IQR)	68 (7.1)	69 (7.5)

### Convergent Validity and Agreement on Travel Mode

**Figure 2:**
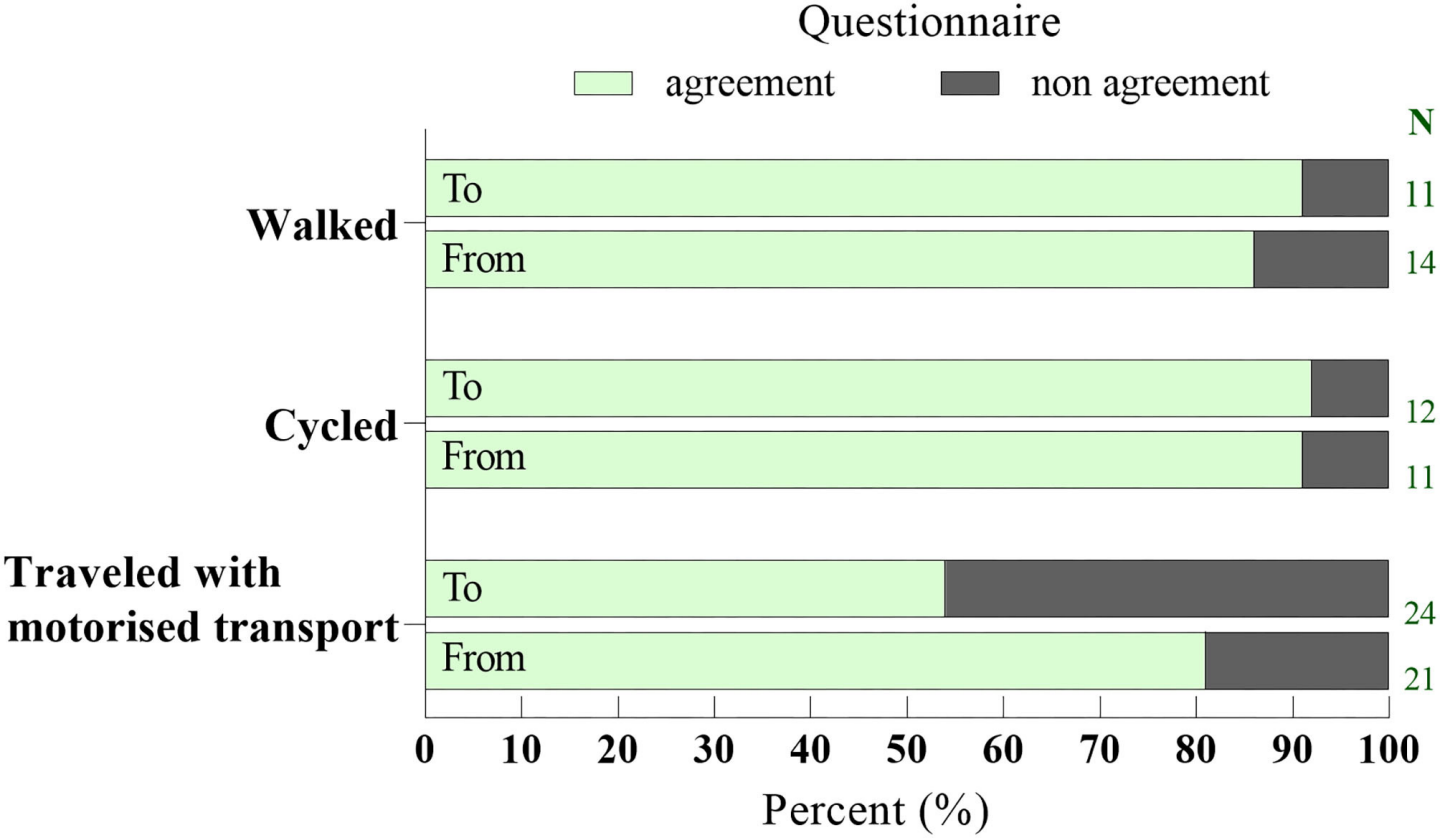
Percentage of reported transport modes to (*n* = 47) and from (*n* = 46) school in the questionnaire, stratified by transport mode from travel diaries.

The analysis of convergent validity showed a kappa coefficient of κ = 0.63, *p* < 0.001 and κ = 0.77, *p* < 0.001 to and from school, respectively. Overall, the agreement between the HBSC questionnaire and the travel diary was 78%. However, after stratification by the typical mode of transport reported in the diary to and from school, motorized transport (67%) had a lower agreement than both walking (88%) and cycling (91%), as illustrated in Figure 2. With respect to trip direction, the agreement for motorized transport was higher from school (54 and 81% to and from school, respectively).

### Convergent Validity and Agreement on Travel Time

**Figure 3:**
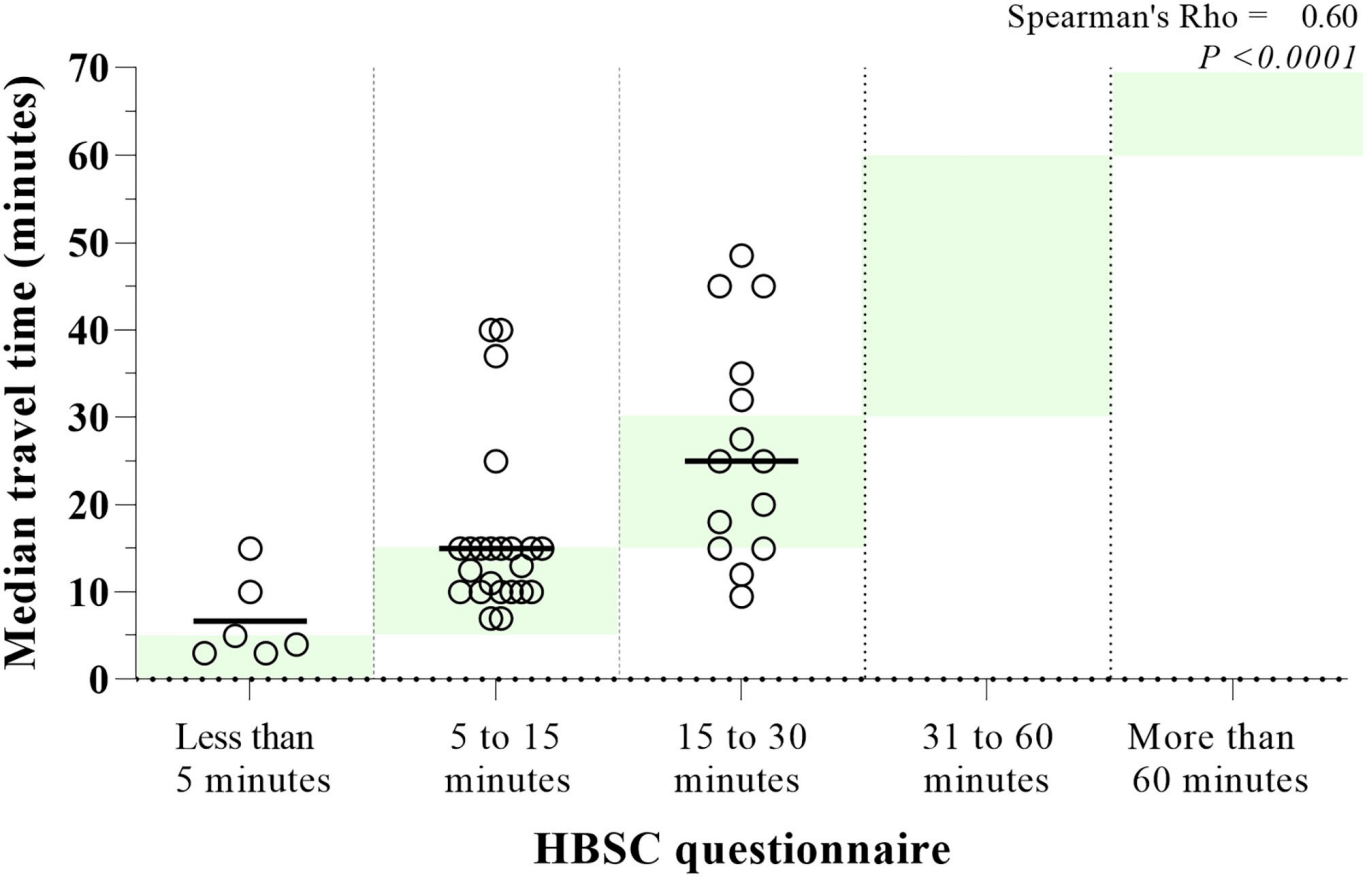
Typical travel duration (median) to school logged in travel diaries (dot) categorized by each participants response from the HBSC questionnaire. Green shadow background indicates the categorize from HBSC in the graph. Vertical line = median in each questionnaire response.

The analysis of convergent validity on travel time showed a Spearman coefficient of ρ = 0.60, *p* < 0.001 to school (Figure 3). Overall, the findings showed a 67% agreement between median travel time in the diary and that reported in the HBSC questionnaire. However, after stratification by the typical travel mode reported in the diary, active commuters had an 86% agreement, while motorized commuters only had a 55% agreement.

## Discussion

Overall, most (78%) participants reported the same mode of transport in the HBSC questionnaire and the diary. However, the level of agreement varied by mode of transport as there was only 54 and 81% agreement among participants reporting motorized travel mode to and from school, respectively. The remaining participants mainly reported cycling or walking instead. Regarding trip direction, a higher agreement was observed for travel from school, and stratification analysis indicated that this difference was most prevalent among motorized commuters. As mentioned earlier, among motorized commuters no participant was driven from school, as opposed to 10 participants to school, as reported in the diary. Differences in the agreement for trip direction can partly be explained by differences in the use of a car.

With respect to travel time, the overall agreement was 67%, which was lower than that for the mode of transport. Moreover, stratification analysis indicated that active commuters more accurately estimated travel time, since the agreement was higher among participants typically traveling by active modes of transport than motorized modes of transport, as reported in the diary. Furthermore, the participants seemed to underestimate travel time in the questionnaire compared to that in the diary (Figure 2), which could be influenced by the lower agreement on travel mode among motorized commuters. According to prior research, it may be more difficult to estimate travel time than the mode of transport. Everson et al. (2019) found higher validity for categorical questions than for items requiring children to estimate time, which they further discussed might be due to the burden of recall.

Our results suggest a kappa coefficient of 0.63 and 0.77 between the HBSC and the diary. Landis and Kock's guidelines define kappa between 0.61 and 0.80 as substantial (Landis and Koch, 1977). However, this classification has been considered arbitrary (Warrens, 2015) and some argue that the cut-off values lack foundation (Vach, 2005).

Petrunoff et al. (2013) conducted a validation study among 45 adults, comparing a diary to an online survey in which the participants answered the following question: “how did you travel for work this week?” Based on a Cohen's kappa of 0.62 when comparing the mode of transport, the instrument was considered to be valid. Their findings were similar to those of the present study. The present study included adolescents and aimed to measure the usual travel mode, whereas Petrunoff et al. included adults and aimed to measure the travel mode during a specific week. Petrunoff et al. found a weaker association for days further back in time than for the most recent days (Petrunoff et al., 2013), which indicates that time between two measurement methods is influenced by recall bias.

Discrepancies between the two measurement methods assessed in the present study may also be explained by social desirability bias (Klesges et al., 2003), as environmentally friendly and physically active modes of transport may be perceived as desirable and may contribute to overreporting of active modes of transport. Another factor that may lead to inconsistencies between the HBSC questionnaire and the diary is estimation bias, particularly if the participants use various travel modes during the school year. The questions “on a typical day is the main part of your journey to school made by…?” and “how much time does it usually take you to get from home to school” were retrieved from the HBSC questionnaire. These questions are broad and depend on the participants' perception of the term “typical” or “usual.” Another point to consider is that we ask for a typical mode or travel time, assuming that the participants have one residence, not including separate homes, with two travel routes, as an option. Furthermore, we compared the HBSC questionnaire items measuring usual travel mode with day-to-day reports. The HBSC questionnaire responses may be affected by the participants' intentions; for instance, a participant may have intentions to cycle to school and report it as the usual travel mode, but due to circumstantial factors, motorized transport could have been used.

### Strengths and Limitations

We compared the HBSC items to a five-day travel diary. One study strength is the inclusion of a diary as opposed to a parental questionnaire, as questionnaires may be less valid than diaries and logs (Bakker et al., 2020).

We included a mention of time and season as the travel mode during summer may differ from that during winter (Liu et al., 2017), and items aimed to measure travel modes in the summer season were included in the present study. Since we modified the HBSC questionnaire items, the comparability between studies is limited. Moreover, long questionnaires may increase participant burden and the risk of careless responding (Rolstad et al., 2011; Bowling et al., 2020); nevertheless, the items included in the present study appeared early in the questionnaire.

One limitation of the present study is the small sample size caused by the low participation rate (Table 1), which may be explained by participant burden, as the participants completed a series of physical tests in the pre-, mid-, and post-data collection phases of the main project.

The HBSC questionnaire asks the participants to report travel mode on a typical day, whereas the diary captures the travel mode during a specific week, assuming that the reported week was typical for every participant. The diary also provides continuous data on travel time, whereas the HBSC questionnaire provides categorical data. Furthermore, there is a lack of temporality between the two estimates of travel mode and time, as the participants answered the HBSC questionnaire items between 3 and 36 days before logging the diary. The lack of agreement might then be attributable to true differences in travel mode/time between the measurement periods. Then again, avoiding holidays and days off at school when distributing the diary would increase the chance of having a typical week, making the diary more comparable to the questionnaire items.

We included Norwegian adolescents in the 9th grade and obtained data for the summer half of the year; hence the findings may not be transferable to other subgroups or seasons. Moreover, the diary has not been previously validated using an objective measure.

This is the first study aiming to validate the HBSC items on active travel and therefore would be relevant for any researchers applying these items as a measurement method. Knowledge about the accuracy of the HBSC questionnaire is essential for researchers and readers. It contributes to a broader understanding of data based on the widely used HBSC items and highlights that the validity may vary among participants traveling with different modes of transport. However, it is uncertain how transferable the findings are to other populations around the world. Differences in climate (Liu et al., 2017), infrastructure and travel habits (Tremblay et al., 2016) can be among factors that limit generalizability to other countries. Nevertheless, our findings can be a supplement to future validation studies and indicate how validity between different populations may vary. Furthermore, more studies are needed on the convergent validity of the HBSC questionnaire items on active travel preferably comparing with observations or objective measures.

## Conclusion

Although the overall agreement between the HBSC questionnaire and the diary was 78%, the questionnaire may underestimate the prevalence of motorized transport compared to that of active modes of transport.

## Data Availability Statement

The data that support the findings of this study are available from the corresponding author, LM, upon reasonable request.

## Ethics Statement

The studies involving human participants were reviewed and approved by the Regional Committees for Medical and Health Research Ethics (REC). However, the need for approval was waived and it was reasoned that our research is not health research. Furthermore, the Norwegian Center for Research Data (project nr.: 49094) assessed the project and concluded that the processing of personal data is regulated by law, §7–27 in the Personal Data Regulations. Written informed consent to participate in this study was provided by the participants' legal guardian/next of kin.

## Author Contributions

LM, TH, and SB conceptualized the aims of the paper. LM drafted the manuscript, designed the diary, and contributed to the data collection. EK, TH, and SB conceived and designed the study design. BH contributed to the data analysis and interpretation of results. All authors have contributed to drafting and critically revised the work. All authors read and approved the final version of the manuscript.

## Funding

The Norwegian Directorate for Education and Training funded the main project, the School in Motion study. The present study was a part of a Ph.D. project and funded by the University of Agder.

## Conflict of Interest

The authors declare that the research was conducted in the absence of any commercial or financial relationships that could be construed as a potential conflict of interest.

## Publisher's Note

All claims expressed in this article are solely those of the authors and do not necessarily represent those of their affiliated organizations, or those of the publisher, the editors and the reviewers. Any product that may be evaluated in this article, or claim that may be made by its manufacturer, is not guaranteed or endorsed by the publisher.
